# Psychometric Properties of the Italian Version of the Short-Form Supportive Care Needs Survey Questionnaire (SCNS-SF34-It): A Multicenter Validation Study

**DOI:** 10.3390/nursrep14010023

**Published:** 2024-01-27

**Authors:** Anita Zeneli, Paolo Leombruni, Marco Miniotti, Emanuela Scarpi, Marco Maltoni, Sara Cavalieri, Valentina Legni, Cristina Nanni, Mihaiela Tarca, Michela Rustignoli, Sandra Montalti

**Affiliations:** 1 Nursing and Technical Directorate, IRCCS Istituto Romagnolo per lo Studio dei Tumori “Dino Amadori” (IRST), Via Maroncelli 40, 47014 Meldola, Italy; anita.zeneli@irst.emr.it (A.Z.); sara.cavalieri@irst.emr.it (S.C.); valentina.legni@irst.emr.it (V.L.); cristina.nanni@irst.emr.it (C.N.); mihaiela.tarca@irst.emr.it (M.T.); michela.rustignoli@irst.emr.it (M.R.); sandra.montalti@irst.emr.it (S.M.); 2 “Rita Levi Montalcini” Department of Neuroscience, University of Turin, Via Cherasco 15, 10126 Turin, Italy; paolo.leombruni@unito.it (P.L.); marco.miniotti@unito.it (M.M.); 3 Unit of Biostatistics and Clinical Trials, IRCCS Istituto Romagnolo per lo Studio dei Tumori “Dino Amadori” (IRST), Via Maroncelli 40, 47014 Meldola, Italy; 4 Department of Medical and Surgical Sciences (DIMEC), University of Bologna, Via Massarenti 9, 40138 Bologna, Italy; marcocesare.maltoni@unibo.it

**Keywords:** cancer patients, short-form supportive care needs survey questionnaire (SCNS-SF34), psychometric property evaluation, validation study

## Abstract

This study aimed to evaluate psychometric properties of the Italian version of the Short-Form Supportive Care Needs Survey Questionnaire (SCNS-SF34) in a cancer population. A multicenter prospective observational study was carried out in outpatient and inpatient settings. The evaluated psychometric properties were as follows: the five-domain structure, the internal consistency, the convergent validity with the Edmond Symptom Assessment System (ESAS) questionnaire, the discriminant validity and test–retest reliability. A total of 714 patients with different types, stages and treatment settings of cancer were recruited. A total of 56% of participants were women, the median age 59 years (range 18–88). The prevalence of patients reporting at least one unmet need was 78.7%. The factor analysis explained 71.3% of the total variance, confirming the five-domain structure of the original model. Internal consistency was good, with Cronbach’s alpha values ranging from 0.87 (“psychosocial need”, “patient support and health system”, “information”) to 0.90 (“sexuality”). The convergent validity of the SCNS-SF34-It with the ESAS scale was low, suggesting that these questionnaires cover different concepts. The SCNS-SF34-It was able to discriminate differences between groups, and the test–retest reliability was good (ICC 0.72–0.84). The SCNS-SF34-It proved to be a reliable instrument for use in clinical practice for evaluating unmet needs in the Italian population of cancer patients. This study was not registered.

## 1. Introduction

Patients affected by cancer experience health problems during the course of their disease [1,2,3,4,5,6]. According to the European Society of Medical Oncology (ESMO), the term “supportive care” refers to the set of all the general and specialty services that are necessary to support cancer patients and their families in order to maintain a multidisciplinary approach in response to the person’s needs at all times throughout the disease treatments. Supportive care aims to optimize patient health outcomes, comfort and functionality, as well as to provide social support to patients and their families [7]. The latest position paper of ESMO advocates for patient-centered care to be integrated by a multidisciplinary team (MDT) to anticancer treatment, from the time of diagnosis and throughout the course of the disease, including end-of-life and survivorship care [8]. This care approach can only be achieved through careful consideration of the patient’s needs, early treatment, control and monitoring of adverse events, physical and psychological assessments and coping of patients with the oncological diseases in all their stages. To achieve these goals, it is necessary to have validated self-reported instruments capable of measuring the patient’s perception of their needs being satisfied. The use of patient-reported outcomes (PROs) in clinical practice is highly encouraged, as it is shown to be associated with better quality of life (QoL), fewer hospitalizations and even increased survival compared with the usual care. Furthermore, measuring the perception of the patient’s need satisfaction provides useful information for designing patient-centered services and implementing individually tailored interventions, as well as for research purposes [9,10]. Several PRO questionnaires specifically designed to evaluate the supportive care needs of cancer patients are now available in different countries. The Supportive Care Needs Survey, in its short form comprising 34 items (SCNS-SF34), has been translated and validated in many languages, and it has showed very good psychometric properties in different cancer populations and countries [11,12,13,14,15,16,17,18,19,20,21,22,23,24,25]. A previous study translated and pre-tested the linguistic and cultural validity of the Italian version of the SCNS-SF34 in a small Italian population. Although the pretest study showed very good psychometric properties of the instrument, it is necessary to validate it on a larger population before introducing it in clinical practice [26].

The study aimed to evaluate the psychometric properties of the Italian version of the SCNS-SF34 in a general population of patients living with cancers of varying types, stages and treatment settings.

## 2. Material and Methods

### 2.1. Study Design

This was a multicenter, prospective, observational study including three oncologic day hospital units, an oncologic ward and a clinical psychology unit in northern Italy. Informed consent and local Ethics Committee’s approval were obtained.

### 2.2. Study Population

The study population consisted of Italian adult cancer patients who had been in care at the participating centers for at least one month, independently of their cancer type, stage, treatment regimen and care setting, who were capable of providing an informed consent and completing the questionnaire.

A subpopulation of patients enrolled in this study simultaneously participated in the validation study of the melanoma module.

Patients with cognitive or language problems that could compromise their ability to complete the survey were not included in the study.

### 2.3. Study Procedures and Data Collection

Research team members (nurses, oncologists and psycho oncologists) approached and informed consecutive patients accessing the participating units between October 2015 and October 2016. Participants were enrolled after obtaining their informed consent to participate and to use their data for the study. Socio-demographic data were obtained via self-reporting. Information about clinical data (diagnosis, disease site and treatment setting) were extracted from the electronic medical records (EMRs). Inpatients completed the survey at the time of admission, while outpatients completed it on the same day of their healthcare procedures in the planned care settings. Data were anonymized by deleting identification information of participants and were registered in the case report forms (CRFs) by the study investigators.

### 2.4. Instruments

The Italian version of the SNCS-SF34 questionnaire, previously pre-tested [26], was used for the study. The instrument measures the level of unmet needs of cancer patients in the last month and includes 34 questions regarding 5 domains: “Psychological needs” (10 items), “Health system and information needs” (11 items), “Physical and daily living needs” (5 items), “Patient care and support needs” (5 items) and “Sexuality needs” (3 items). Patients are asked to rate the intensity of each need using a 5-point Likert scale, ranging from 1 (absence of need) to 5 (high presence of need): the higher the score, the higher the level of unmet need. The level of unmet needs is divided into two categories: “no need” (scores 1 and 2) and “need” (scores >2); the latter is further divided into “low need” (score 3), “moderate need” (score 4) and “high need” (score 5). Standardized Likert summated scores were used to calculate domain scores ranging from 0 to 100.

To assess the convergent validity of the SCNS-SF34 questionnaire, reporting of the intensity of symptoms measured through the validated Italian version of the Edmonton Symptom Assessment System (ESAS) questionnaire [27] was also carried out. The ESAS included questions regarding the intensity of 9 symptoms (pain, fatigue, nausea, depression, drowsiness, loss of appetite, malaise, dyspnea, anxiety) evaluated with a numerical rating scale ranging from 0 (no symptom) to 10 (highest symptom intensity).

### 2.5. Statistical Analysis

Continuous variables were summarized by descriptive statistics (mean, standard deviation—SD, median, minimum and maximum), and categorical variables were summarized using counts of patients and percentages. According to the guide of the SCNS-SF34-It questionnaire scoring and analysis, when missing data occurred and when less than half of the items on a domain were missing, a value equal to the average of the other scores on that domain was imputed [28].

**Factor validity** was evaluated by a confirmatory factor analysis to verify whether the original five-factor model of the SCNS-SF34 could be replicated in the Italian version. Principal component analysis with varimax rotation was used with eigenvalues >1.0 (Kaiser–Guttman’s criterion). Criteria for goodness-of-fit indices were Root Mean Square Error of Approximation (RMSEA) <0.06, Comparative Fit Index (CFI) and Tucker–Lewis Index Non-Normed Fit Index (TLI-NNFI) ≥0.90. These values are indicative of a good fit between the hypothesized model and the observed data. Appropriateness of principal component analysis was examined by Bartlett’s test of sphericity (*p* < 0.05) and the Kaiser–Meyer–Olkin index of sampling adequacy (KMO) ≥0.60 [29].

**Internal consistency** was assessed using Cronbach’s alpha coefficient. A value between 0.70 and 0.90 was considered good and demonstrates satisfactory homogeneity of the instrument [30].

The **convergent validity** was explored by calculating Pearson’s correlation coefficient between the SCNS-SF34-It domain and the ESAS scale scores reported by patients. We hypothesized that patients with a higher score of symptoms were expected to have higher needs. We considered weak correlation values of Pearson coefficient below 0.30, moderate correlation r = 0.30–0.50 and strong correlation r > 0.50 [13].

The **discriminant validity**, that is, the ability of the SCNS-SF34-It to discriminate between subgroups of patients, was also investigated. We expected that (1) females would report a higher level of need on the physical and daily living and psychological domain; (2) younger patients (<66 years) would report a higher level of need than older patients (>66 years) on all domains except for physical and daily living; (3) patients living alone would report a higher level of need than patients living with others; (4) patients in a metastatic setting of their disease would report a higher level of need than patients in other settings. These hypotheses were examined by Student’s T test or analysis of variance (ANOVA).

Intraclass correlation coefficients (ICC) was used to analyze **test–retest reliability**. For this purpose, we repeated the survey nearly one month after the first self-administration in 22% of randomly selected participants. An ICC value ≥0.70 was considered good [30].

The presence of **floor or ceiling effects** were investigated using frequency tables. Floor or ceiling effects were considered to be present if >50% of patients achieved the lowest or highest possible scores, respectively [31].

Sample size was based on a rule of thumb of 20 subjects for each item of the original scale (34 items). At least 680 patients were needed for factor analysis, 50 patients for constructed validity analysis and 50 patients for test–retest reliability [32].

Based on previous surveys conducted at our center, we expected a response rate of 88%. Thus, considering that about 12% of patients would have not completed the questionnaire, a further 84 patients were enrolled for a total of 764 patients.

A subcohort of at least 200 patients affected by melanoma was included in this population because the validation study of the melanoma module was conducted simultaneously [33]. All tests were two-tailed at a significance level of 0.05. Statistical analyses were performed using SAS Statistical software version 9.4 (SAS Institute, Cary, NC, USA).

## 3. Results

### 3.1. Patients Characteristics

We approached a total number of 950 patients, and obtained informed consent to participate in the study from 764 patients. Out of them, 714 correctly completed the questionnaire (93.5%) while the data of the remaining 50 participants was not evaluable because of a lot of missing information. Characteristics of study participants are presented in Table 1.

The median age of the study population was 59 years (range 18–88) and 402 (56.3%) were female; 87.7% of the patients had a secondary or higher education level. The majority of patients were married (67.1%), living with a partner/someone (83.9%), employed (48.7%), with metastatic disease stage (64.5%) and undergoing outpatient treatment (82%).

### 3.2. Missing Data

Missing data rate of the SCNS-SF34-It questionnaire ranged from 1.0% to 5.2% per item (Table 2).

Respondents and non-respondents did not significantly differ regarding socio-demographic and clinical characteristics. However, missing data were more frequent for patients with metastatic cancer (about 73% for all items), age >56 years (81% for item 11, 93% for item 12, 87% for items 15 and 16, 89% for item 28), males (75% for item 9, 71% for item 10, 68% for item 28), primary education level (46% for items 6 and 15) and genitourinary tract cancer (41% for item 25) (*p* < 0.05). No other significant differences were found.

The average time to complete the survey was about 15 min (SD ± 8.5; min = 2; max = 60). Among the 714 evaluable questionnaires, it was reported in only 27 (3.8%) of them that patients asked for help during the survey completion. The main reasons for needing help were the physical limitations (for example, the presence of medical devices limiting the use of the right hand, i.e., access devices, plegias or other physical limitations).

### 3.3. Factor Analysis

The results of the confirmatory factor analysis replicated the original five-factor structure of SNCS-SF34 in our study population. The examination of residuals of the initial model showed a pair of items with higher correlations within the same factor (items 15 and 16, r = 0.82), suggesting redundant semantical content. Thus, the initial model was modified, allowing the correlations between residuals of redundant pair of items within the same factor (correlations between 0.07 and 0.35). Indicators of fit of this modified model were slightly improved, with RMSEA = 0.06, CFI = 0.87 and Tucker–Lewis index = 0.91. The KMO statistic was 0.95 and Chi-squared test was 19,083.60, *p* < 0.001. The factor analysis indicated a five-factor solution, accounting together for 71.3% of the total variance. Factor 1 (Health system and information needs) comprises 11 items and explained 24.8% of the total variance. Ten items concerning psychological needs loaded on Factor 2, accounting for 19.9% of total variance. Five items related to physical and daily living needs loaded on Factor 3 (11.3% of total variance) and five more items to assess needs related to patient care and support loaded on Factor 4 (8.3% of total variance). The remaining three items addressing sexual relationship needs were loaded on Factor 5, accounting for 7%. Item to total score correlation coefficients for all items exceeded 0.50, with high correlation between “Health system and information” and “Patient care and support” need scales (r = 0.75) as well as between “Physical and daily living” and “Psychological need” scales (r = 0.60). Therefore, the original factor structure was considered in this study. Table 2 shows results derived from confirmatory factor analysis.

### 3.4. Prevalence of Psychosocial Unmet Needs

The overall prevalence of patients reporting at least one unmet need defined as a score ≥ 3 on the 5-point Likert scale was 78.7%. Details about unmet needs for each item are shown in Table 2. The top five unmet needs were “Uncertainty about the future” (47.8%), “Concerns about the worries of these close to you” (46.8%), “Fears about the cancer spreading” (45.7%), “Lack of energy” (44.1%) and “Anxiety” (36.1%). The prevalence of unmet needs were related to the domains of “Psychological needs” and “Physical and daily living”.

### 3.5. Internal Consistency/Reliability

The descriptive statistics and internal consistencies of the SCNS-SF34-It domains are reported in Table 3.

The highest mean values were achieved on the “Health system and information” and “Physical and daily living” domains. Internal consistency as evaluated with Cronbach’s alpha values ranged from 0.87 (“Psychosocial need”, “Patient care and support”, “Health system and information”) to 0.90 (“Sexuality”).

In all domains, less than 50% of patients obtained the lowest (0) or the highest (100) scores, suggesting the absence of floor or ceiling effects. The lowest scores ranged from 9.5% (“Psychological need”) to 49.2% (“Sexuality”) while the highest ranged from 0.1% to 1.3%.

### 3.6. Test–Retest Reliability

The test–retest reliability of the SCNS-SF34-It domain was good, with ICC ranging from 0.72 for “Patient care and support” to 0.84 for “Psychosocial need”. “Sexuality” showed an ICC of 0.78, “Health system and information” an ICC of 0.80 and “Physical and daily living” an ICC of 0.81.

### 3.7. Convergent Validity

Results of correlation between SCNS-SF34-It subscales and the ESAS scale are shown in Table 4.

The expected hypothesis is that the unmet needs and the related subscales are related to the intensity of the symptoms. Most of the ESAS symptoms did not correlate highly with SCNS-SF34-It domains, suggesting that these questionnaires cover different concepts. However, we found significant but weak positive associations between total Symptom Distress Score and the “Physical and daily living”, “Psychological” and “Patient care and support” subscales (r = 0.27, r = 0.19 and r = 0.16, respectively). Weak positive correlations were also found between fatigue and drowsiness and the “Physical and daily living” subscale of the SCNS-SF34-It questionnaire (r = 0.25 and r = 0.20, respectively). Drowsiness, anxiety and depression were found to be correlated with the “Psychological needs” domain (r = 0.20, r = 0.19 and r = 0.19) while anxiety was found to be correlated with the “Patient care and support” (r = 0.17) and “Health system and information” domains (r = 0.16).

### 3.8. Discriminant Validity

Results about discriminant validity showed statistically significant differences in general unmet needs and patient characteristics (Table 5).

This was true for the age, marital status, cancer site and setting. Women reported higher scores for levels of need in “physical and daily living” and “psychological” domains than men (mean value 27.3 vs. 20.5, *p* < 0.0001; 32.1 vs. 26.4, *p* = 0.002, respectively). Unemployed patients reported higher levels of needs in all domains of the SCNS-SF34-It while patients without a partner reported higher levels of need in different domains of the SCNS-SF34-It. As for the pathology, patients with pulmonary pathology presented higher levels of “need” in almost all domains. Elderly people (≥75 years) reported higher needs in all survey domains expect for sexuality, of which the level of need was the lowest compared to all the other age groups (mean value 11.1 vs. 16.7; 16.6 and 18.0, *p* < 0.0001). Moreover, patients with depression, anxiety and drowsiness (intensity of symptom >0 was considered as “present”) reported higher score levels in all domains.

## 4. Discussion

Since the Italian version of the SCNS-SF34 was adapted and pre-tested in a small population, the evaluation of its psychometric properties in a larger cancer population was necessary. With a multicenter study design, we validated the Italian version of the SCNS-SF34 in a general population of patients with heterogeneous cancer diseases and treatment settings. Demographic characteristics of patients involved in our study were similar to those of the previous validation studies that tested the instrument in general cancer populations in other countries [12,16,34]. Even though the completion time results were slightly higher compared to the original version (15 vs. 10 min), the high response rate (93.5%) and the low missing data in the evaluated questionnaires (1.0% to 5.2% per item) suggest that the Italian version of the SCNS-SF34 is well accepted and easy to self-administer [12]. The factor analysis performed with data observed in our study showed a good fit with the five-domain structure proposed in its original version by confirming its validity when used in Italian cancer patients [12]. This result suggests that the original conceptualization of the instrument applies to the Italian context, too. In contrast, the five-domain structure of the same instrument was modified in the validation studies of Chinese and Turkish versions because some items did not load under their original domain [14,18,20]. Such differences may be explained by the cultural differences and the characteristics of supportive care services among countries.

Consistently with previous studies, the Cronbach’s alpha values ranged from 0.87 (“Psychosocial needs”, “Patient care and support”, “Health system and information”) to 0.90 (“Sexuality”), showing good internal consistency of the SCNS-SF34-It, confirming its reliability in a larger Italian cancer population [12,13,14,15,16,17,18,19,20,21,22,23,24,25,34]. The statistically significant differences found in our study regarding general unmet needs and patient characteristics proves that the SCNS-SF34-It is capable of discriminating the perceived needs according to patients’ characteristics, i.e., age, sex, marital and employment status, cancer site and disease setting. While age- and sex-related differences in perceived needs in specific domains of the SCNS-SF34 were proved in most if not all of the previous validation studies [12,13,14,15,16,17,18,19,20,21,22,23,24,25,34], differences in perceived needs related to disease stage were not observed in the German population. The authors stated that this finding could be explained by the fact that all their participants were receiving active cancer treatments [16]. The most frequent unmet needs in our study population were related to the “Psychological needs” and “Physical and daily living” domains. The top five perceived needs in our population were “*Uncertainty about the future*” (47.8%), “*Concerns about the worries of those close to you*” (46.8%) and “*Fears about the cancer spreading*” (45.7%), followed by “*Lack of energy*” (44.1%). The distribution of unmet needs found in our study is similar but not equivalent to the findings of previous studies. We believe that a full comparison of the prevalence of unmet needs could only be possible for similar patient populations and for centers offering similar supportive care services.

In all domains, <50% of patients obtained either the lowest (0) or highest (100), suggesting the absence of floor or ceiling effects. This result was in line with the original version of the SCNS-SF34 validation study [12].

With the ICC ranging from 0.72 to 0.82, the SCNS-SF34-It proved its good test–retest reliability, demonstrating the stability of the instrument over time.

Unlike the previous validation studies that used the Health-related quality of life (EORTC QLQ-C30), the Hospital Anxiety and Depression Scale (HADS) and Distress Thermometer (DT) scales for evaluating the convergent validity of the SCNS-SF34, we evaluated the convergent validity of the SCNS-SF34-It with the ESAS scale. We expected the unmet need subscale scores to be related to the intensity of symptoms. Most of the ESAS symptoms did not highly correlate with SCNS-SF34-It domains. However, weak but positive associations were observed in our population between the total Symptom Distress Score and some of the SCNS-SF34-It domains (“Physical and daily living”, “Psychological needs” and “Patient care and support” subscales: r = 0.27, r = 0.19 and r = 0.16, respectively). This finding shows that these questionnaires cover different concepts, suggesting that the SCNS-SF34 adds new information to other assessments made by using self-reported instruments in clinical practice. In fact, while the self-reported ESAS scale evaluates the level of symptoms perceived by the patient in a 24–48 h timeline, the SCNS-SF34 very likely measures the level of needs for support to deal with such health problems during the last 30 days from the moment of completing the survey. This is reinforced by the fact that we observed that, even though some patients reported low level of symptoms with the ESAS score, they indicated high levels of needs in some domains of the SCNSC-SF34-It. Thus, the instrument is useful for evaluating the patient needs and for implementing tailored interventions.

### Strengths and Limitations

Our study presents some limitations that need to be mentioned to allow readers to decide whether our results apply to their own context. The main limitation is related to the fact that ours was a multicenter study that included four hospitals located in northern Italy. This may limit the generalizability of the results to the whole territory, since differences in supportive care service availability may occur within the country. The second limitation is related to the study design, which did not allow for evaluation of how the SCNS-SF34-It is capable of predicting changes to unmet needs over time in different moments of disease trajectory.

However, the Italian version of the SCNS-SF34 tested in this study was developed as a result of the cultural adaption process and its content and face validity was previously tested [26].

Furthermore, we believe that the sample size, the low rate of missing data and the fact that we included a heterogeneous population (different primary cancer sites, stages, treatment settings and socio-demographic characteristics) can contribute to validity of our results.

## 5. Conclusions

In conclusion, the SCNS-SF34-It is a valid and reliable tool that we recommend for use in clinical settings to address patient needs and program tailored supportive care services.

## Figures and Tables

**Table 1:** Characteristics of 714 patients with evaluable SCNS-SF34-It questionnaires.

	**N. (%)**
**Age (years)**: median value (range)	59 (18–88)
18–35	35 (4.9)
46–55	246 (34.5)
56–75	388 (54.3)
>75	45 (6.3)
**Gender**	
Male	312 (43.7)
Female	402 (56.3)
**Living**	
Living with others	453 (83.9)
Living alone	87 (16.1)
Unknown	174
**Occupational status**	
Employed	332 (48.7)
Retired	273 (40.0)
Other	77 (11.3)
Unknown	32
**Education level**	
Primary	85 (12.3)
Lower secondary	190 (27.5)
Upper secondary	278 (40.3)
Bachelor/master/doctorate	137 (19.9)
Unknown	24
**Number of children**	
0	82 (17.7)
≥1	380 (82.3)
Unknown	252
**Neighborhood of residence**	
Rural	162 (30.7)
Urban	365 (69.3)
Unknown	187
**Site of disease**	
Breast	141 (19.8)
Gastrointestinal	98 (13.7)
Genitourinary	75 (10.5)
Hematological	70 (9.8)
Melanoma	215 (30.1)
Lung	38 (5.3)
Other	77 (10.8)
**Setting**	
Metastatic	349 (48.9)
Adjuvant	112 (15.7)
Induction	40 (5.6)
Survivor	181 (25.3)
Palliative	32 (4.5)

“Setting” means the type of care setting where the cancer care, relating to the treatment start date (cancer), took place. “Induction” is related to treatment and is the initial chemotherapy a person receives before undergoing additional cancer treatment, such as maintenance chemotherapy, radiation therapy, or surgery. Cancer “Survivors” is refers to individuals who have successfully completed curative treatments or those who have transitioned to maintenance or prophylactic therapy.

**Table 2:** SCNS-SF34-It item characteristics.

			Item Characteristics				Factor						
	Dimensions and Items		Mean	SD	Missing Value N (%)		Health System and Information	Psychological	Physical and Daily Living	Patient Care and Support	Sexuality	Item Total Correlation	% Patients with Unmet Need ^1^
	***Health system and information needs***												
26	Being adequately informed about the benefits and side effects of treatments before you choose to have them		2.12	1.09	23 (3.0)		0.86					0.81	20.3
25	Being given explanations of those tests for which you would like explanations		2.12	1.01	17 (2.2)		0.85					0.80	21.4
29	Being informed about things you can do to help yourself to get well		2.30	1.25	20 (2.6)		0.85					0.84	30.5
28	Being informed about cancer which is under control or diminishing (that is, remission)		2.35	1.27	19 (2.5)		0.84					0.81	29.0
33	Being treated in a hospital or clinic that is as physically pleasant as possible		2.09	1.19	17 (2.2)		0.83					0.81	19.7
23	Being given written information about the important aspects of your care		2.08	1.11	15 (2.0)		0.82					0.77	21.4
27	Being informed about your test results as soon as feasible		2.25	1.19	19 (2.5)		0.82					0.81	24.9
34	Having one member of hospital staff with whom you can talk to about all aspects of your condition, treatment and follow-up		2.27	1.29	18 (2.4)		0.82					0.82	29.0
32	Being treated like a person not just another case		2.01	1.17	22 (2.9)		0.81					0.76	18.3
24	Being given information (written, diagrams, drawings) about aspects of managing your illness and side effects at home		1.99	1.09	21 (2.7)		0.79					0.72	21.3
30	Having access to professional counseling (e.g., psychologist, social worker, counselor, nurse specialist) if you, family or friends need it		1.99	1.19	17 (2.2)		0.77					0.77	24.2
	***Psychological needs***												
11	Uncertainty about the future		2.51	1.39	21 (2.7)			0.80				0.84	47.8
12	Learning to feel in control of your situation		2.10	1.12	15 (2.0)			0.79				0.48	32.6
9	Fears about the cancer spreading		2.50	1.39	12 (1.6)			0.77				0.79	45.7
7	Feeling down or depressed		1.95	1.14	15 (2.0)			0.76				0.74	31.1
8	Feelings of sadness		2.06	1.16	20 (2.6)			0.76				0.76	35.4
14	Feelings about death and dying		2.02	1.24	23 (3.0)			0.74				0.74	32.5
10	Worry that the results of the treatment are beyond your control		2.02	1.23	14 (1.8)			0.73				0.51	32.2
13	Keeping a positive outlook		2.11	1.18	21 (2.7)			0.73				0.74	30.7
6	Anxiety		2.10	1.22	15 (2.0)			0.71				0.73	36.1
17	Concerns about the worries of those close to you		2.52	1.36	19 (2.5)			0.63				0.69	46.8
	***Physical and daily living***												
5	Not being able to do the things you used to do		2.07	1.24	27 (3.5)				0.83			0.81	35.0
4	Work around the home		1.89	1.14	26 (3.4)				0.80			0.77	26.7
2	Lack of energy/tiredness		2.39	1.26	31 (4.1)				0.73			0.84	44.1
3	Feeling unwell a lot of the time		1.73	1.06	21 (2.7)				0.71			0.75	23.4
1	Pain		1.82	1.12	12 (1.6)				0.54			0.66	23.0
	*Patient care and support needs*												
21	Hospital staff attending promptly to your physical needs		1.90	0.96	8 (1.0)					0.89		0.84	14.0
20	Reassurance by medical staff that the way you feel is normal		1.91	0.96	13 (1.7)					0.86		0.83	14.9
22	Hospital staff acknowledging and showing sensitivity to your feelings and emotional needs		1.95	0.99	17 (2.2)					0.79		0.79	16.5
19	More choice about which hospital you attend		1.58	0.95	18 (2.4)					0.66		0.74	12.0
18	More choice about cancer specialists you see		1.66	1.00	17 (2.2)					0.64		0.78	15.0
	***Sexuality needs***												
16	Changes in sexual relationship		1.68	1.12	34 (4.4)						0.95	0.85	20.5
15	Changes in sexual feelings		1.75	1.17	40 (5.2)						0.92	0.87	23.4
31	To be given information about sexual relationships		1.62	1.01	31 (4.1)						0.50	0.73	16.1

Abbreviations: SD, standard deviation. ^1^ Defined as patients who rated ≥3 on the 5-point Likert scale.

**Table 3:** Descriptive statistics and Cronbach’s alpha for each SCNS-SF34-It domain.

Domain	No. of Items	Mean (SD) (0–100)	Median (IQR)	Lowest Score (Floor) No. (%)	Highest Score (Ceiling) No. (%)	Cronbach’s Alpha Coefficient
Physical and daily living	5	29.6 (23.9)	25 (35)	150 (21.0)	1 (0.1)	0.89
Psychological needs	10	24.3 (22.9)	20 (35)	68 (9.5)	2 (0.3)	0.87
Sexuality	3	17.0 (23.7)	8 (25)	351 (49.2)	6 (0.8)	0.90
Patient care and support	5	20.0 (19.9)	15 (20)	153 (21.4)	2 (0.3)	0.87
Health system and information	11	28.5 (24.5)	25 (25)	86 (12.0)	9 (1.3)	0.87

Abbreviations: SD, standard deviation; IQR, inter-quartile range.

**Table 4:** Correlation between SCNS-SF34-It domains and the ESAS scale.

	SCNS-SF34-It Domains									
	Physical and Daily Living		Psychological		Sexuality		Patient Care and Support		Health System and Information	
ESAS Scale	r	*p*	r	*p*	r	*p*	r	*p*	r	*p*
Pain	0.18	0.003	0.14	0.024	0.02	0.689	0.15	0.012	0.06	0.278
Fatigue	0.25	0.0002	0.15	0.025	0.09	0.176	0.11	0.107	0.12	0.070
Nausea	0.08	0.163	0.06	0.316	0.03	0.601	0.07	0.280	0.01	0.837
Depression	0.16	0.006	0.19	0.002	0.10	0.085	0.14	0.018	0.12	0.049
Anxiety	0.18	0.003	0.19	0.001	0.04	0.540	0.17	0.005	0.16	0.007
Drowsiness	0.20	0.001	0.20	0.0009	0.08	0.162	0.14	0.025	0.14	0.016
Appetite	0.06	0.355	0.04	0.511	−0.04	0.489	0.06	0.304	0.04	0.490
Well-being	0.12	0.044	0.12	0.046	−0.06	0.363	0.11	0.077	0.09	0.136
Shortness of breath	0.10	0.084	0.05	0.370	−0.12	0.055	0.04	0.545	0.08	0.189
Symptom Distress Score *	0.27	<0.0001	0.19	0.004	0.04	0.570	0.16	0.019	0.09	0.185

* Total ESAS scores.

**Table 5:** Discriminant validity of SCNS-SF34-It.

	Physical and Daily Living		Psychological		Sexuality		Patient Care and Support		Health System and Information	
Characteristics	Mean Value (SD)	*p* ^1^	Mean Value (SD)	*p* ^1^	Mean Value (SD)	*p* ^1^	Mean Value (SD)	*p* ^1^	Mean Value (SD)	*p* ^1^
**Age (years)**										
18–35	22.9 (19.1)		25.4 (21.5)		16.7 (21.0)		18.1 (11.5)		25.5 (17.7)	
36–55	22.8 (23.8)		29.2 (22.8)		16.6 (22.9)		20.1 (20.8)		27.2 (23.5)	
56–75	24.7 (22.0)		29.7 (24.4)		18.0 (24.5)		19.7 (19.2)		28.9 (24.5)	
>75	30.8 (27.0)	<0.0001 ^2^	34.0 (27.6)	<0.0001 ^2^	11.1 (22.6)	<0.0001 ^2^	23.5 (25.6)	<0.0001 ^2^	34.0 (33.1)	<0.0001 ^2^
**Gender**										
Male	20.5 (21.0)		26.4 (23.2)		17.5 (24.4)		19.6 (20.5)		28.2 (24.6)	
Female	27.3 (23.8)	<0.0001	32.1 (24.2)	0.002	16.7 (23.1)	0.654	20.3 (19.5)	0.620	28.7 (24.5)	0.788
**Education level**										
Primary–lower secondary	26.4 (23.8)		29.6 (25.8)		17.1 (24.3)		21.4 (20.8)		29.7 (25.7)	
Upper secondary–bachelor	22.9 (22.4)	0.051	29.3 (22.7)	0.892	16.5 (23.1)	0.754	18.7 (18.9)	0.080	27.3 (23.4)	0.192
**Marital status**										
Married	22.2 (21.6)		27.8 (22.8)		16.7 (23.2)		19.0 (20.0)		26.9 (23.8)	
Other	29.6 (25.0)	<0.0001	34.2 (26.1)	0.001	17.6 (24.7)	0.665	22.5 (19.8)	0.037	32.5 (26.0)	0.005
**Living**										
With others	25.7 (21.8)		30.9 (23.7)		19.8 (24.7)		21.3 (20.9)		29.2 (24.7)	
Alone	34.5 (26.0)	0.0009	36.6 (26.2)	0.045	15.5 (24.1)	0.135	24.8 (20.0)	0.149	35.2 (26.4)	0.039
**Occupational status**										
Employed	20.7 (22.1)		26.2 (21.5)		16.3 (22.1)		19.0 (18.7)		27.0 (22.7)	
Retired	26.9 (22.3)		31.5 (25.3)		16.6 (23.7)		21.7 (21.2)		30.5 (26.2)	
Other	27.5 (23.9)	<0.0001 ^2^	35.0 (24.9)	<0.0001 ^2^	17.5 (24.6)	<0.0001 ^2^	19.1 (20.9)	<0.0001 ^2^	29.2 (25.8)	<0.0001 ^2^
**Number of children**										
0	27.3 (22.9)		28.1 (20.1)		19.4 (23.5)		19.9 (16.8)		28.6 (22.3)	
≥1	26.1 (23.0)	0.678	31.4 (24.6)	0.261	17.2 (24.5)	0.459	19.9 (17.8)	0.995	27.9 (24.9)	0.813
Site of disease										
Breast	28.3 (23.0)		33.8 (24.4)		19.8 (24.1)		22.0 (20.8)		27.6 (24.3)	
Gastrointestinal	27.9 (21.5)		31.6 (22.6)		16.3 (21.8)		21.6 (19.2)		32.4 (25.0)	
Genitourinary	27.7 (23.9)		27.0 (23.0)		19.4 (25.6)		22.7 (22.0)		32.0 (26.4)	
Hematological	26.1 (23.1)		32.3 (26.3)		19.0 (24.9)		21.7 (20.5)		34.5 (26.1)	
Melanoma	17.0 (21.3)		24.0 (21.8)		11.5 (19.4)		14.4 (16.1)		22.7 (21.3)	
Lung	33.8 (25.5)		38.7 (27.6)		19.1 (29.5)		24.9 (25.3)		33.6 (30.5)	
Other	23.5 (21.2)	<0.0001 ^2^	30.5 (23.9)	<0.0001 ^2^	23.2 (27.2)	<0.0001 ^2^	23.3 (20.8)	<0.0001 ^2^	30.1 (23.4)	<0.0001 ^2^
**Setting**										
Metastatic	28.1 (23.0)		33.7 (24.6)		20.8 (25.5)		22.3 (21.2)		30.3 (25.4)	
Adjuvant	26.5 (22.2)		29.6 (23.1)		15.3 (21.1)		20.5 (18.6)		29.0 (24.5)	
Induction	24.6 (21.5)		31.2 (24.4)		20.0 (28.3)		22.4 (23.4)		34.0 (28.0)	
Survivor (follow-up)	19.7 (24.0)		14.4 (28.8)		15.6 (23.3)		20.6 (21.1)		21.9 (16.1)	
Palliative	24.7 (25.6)	<0.0001 ^2^	27.4 (22.7)	<0.0001 ^2^	17.2 (24.0)	<0.0001 ^2^	20.9 (20.8)	<0.0001 ^2^	30.4 (23.4)	<0.0001 ^2^
**ESAS scale**										
Pain: present	37.1 (20.3)		40.0 (23.0)		20.6 (28.7)		25.7 (18.8)		31.5 (22.8)	
absent	26.5 (21.6)	0.007	31.0 (22.7)	0.032	18.9 (25.1)	0.712	19.6 (20.6)	0.101	29.8 (26.0)	0.711
Fatigue: present	32.9 (21.9)		34.7 (22.5)		21.3 (27.3)		22.5 (22.4)		33.8 (28.7)	
absent	22.9 (20.8)	0.0008	28.4 (21.7)	0.038	16.5 (24.2)	0.177	17.2 (17.7)	0.049	25.4 (22.5)	0.014
Nausea: present	37.8 (23.0)		36.9 (11.3)		24.0 (31.0)		26.2 (21.2)		29.8 (24.7)	
absent	27.3 (21.5)	0.176	32.2 (23.2)	0.569	18.9 (25.5)	0.586	20.1 (20.4)	0.406	30.2 (25.9)	0.971
Depression: present	42.9 (20.7)		52.2 (22.9)		30.4 (29.9)		33.5 (22.3)		44.5 (30.1)	
absent	26.9 (21.4)	0.007	31.3 (22.4)	0.0008	18.6 (25.3)	0.093	19.7 (20.2)	0.014	29.6 (25.5)	0.036
Anxiety: present	45.5 (22.0)		53.9 (23.9)		21.8 (24.7)		37.7 (24.1)		53.8 (33.1)	
absent	26.8 (21.3)	0.002	31.3 (22.4)	0.0005	19.0 (25.7)	0.706	19.6 (19.9)	0.002	29.2 (25.1)	0.0007
Drowsiness: present	53.1 (15.8)		59.8 (15.4)		36.5 (35.6)		40.2 (26.2)		54.8 (28.8)	
absent	26.8 (21.2)	0.0006	31.4 (22.6)	0.0005	18.6 (25.2)	0.052	19.7 (20.0)	0.005	29.4 (25.5)	0.006
Appetite: present	30.8 (20.2)		33.5 (17.3)		14.8 (23.5)		23.9 (20.1)		33.1 (27.9)	
absent	27.4 (21.7)	0.523	32.2 (23.3)	0.824	19.4 (25.7)	0.464	20.1 (20.5)	0.444	29.9 (25.8)	0.620
Well-being: present	37.4 (24.5)		43.1 (26.2)		20.6 (34.8)		28.9 (24.8)		38.8 (28.6)	
absent	26.7 (21.1)	0.028	31.4 (22.4)	0.024	19.1 (24.8)	0.792	19.7 (20.0)	0.048	29.6 (25.7)	0.119
Shortness of breath: present	46.2 (21.0)		40.3 (19.4)		20.7 (33.6)		20.0 (17.1)		42.0 (29.7)	
absent	27.3 (21.4)	0.008	32.1 (23.0)	0.480	19.4 (25.7)	0.483	20.3 (20.6)	0.975	30.0 (25.9)	0.356
Symptom Distress Score ^3^: present	32.6 (21.6)		34.8 (22.3)		19.7 (26.4)		22.9 (22.3)		32.1 (27.9)	
absent	22.3 (20.9)	0.0004	27.3 (21.5)	0.012	17.5 (24.9)	0.517	16.3 (17.1)	0.013	25.4 (22.5)	0.048

Abbreviations: SD, standard deviation; ANOVA, analysis of variance. ^1^ *t*-test; ^2^ ANOVA; ^3^ Total ESAS scores.

## Data Availability

The datasets generated and/or analyzed during the current study are available from the corresponding author on reasonable request.

## Public Involvement Statement

No public involvement in any aspect of this research.

## Guidelines and Standards Statement

https://www.equator-network.org/reporting-guidelines/recommendations-for-reporting-the-results-of-studies-of-instrument-and-scale-development-and-testing/ (accessed on 25 January 2024).

## References

[1] Carey M., Lambert S., Smits R., Paul C., Sanson-Fisher R., Clinton-McHarg T. (2012). The unfilled promise: A systematic review of interventions to reduce the unmet supportive care needs of cancer patients. Support. Care Cancer.

[2] Thorsen L., Gjerset G.M., Loge J.H., Kiserud C.E., Skovlund E., Fløtten T., Fosså S.D. (2011). Cancer patients’ needs for rehabilitation services. Acta Oncol..

[3] Li W.W.Y., Lam W.W.T., Au A.H.Y., Ye M., Law W.L., Poon J., Kwong A., Suen D., Tsang J., Girgis A. (2012). Interpreting differences in patterns of supportive care needs between patients with breast cancer and patients with colorectal cancer. Psychooncology.

[4] Beesley V.L., Price M.A., Webb P.M., O’Rourke P., Marquart L., Butow P.N., Australian Ovarian Cancer Study Group (2013). Changes in supportive care needs after first-line treatment for ovarian cancer: Identifying care priorities and risk factors for future unmet needs. Psychooncology.

[5] Boyes A.W., Girgis A., D’Este C., Zucca A.C. (2012). Prevalence and correlates of cancer survivors’ supportive care needs 6 months after diagnosis: A population-based cross-sectional study. BMC Cancer.

[6] Jorgensen M.L., Young J.M., Harrison J.D., Solomon M.J. (2012). Unmet supportive care needs in colorectal cancer: Differences by age. Support. Care Cancer.

[7] Hui D., De La Cruz M., Mori M., Parsons H.A., Kwon J.H., Torres-Vigil I., Kim S.H., Dev R., Hutchins R., Liem C. (2013). Concepts and definitions for “supportive care”, “best supportive care”, “palliative care” and “hospice care” in the published literature, dictionaries, and textbooks. Support. Care Cancer.

[8] Jordan K., Aapro M., Kaasa S., Ripamonti C., Scotté F., Strasser F., Young A., Bruera E., Herrstedt J., Keefe D. (2018). European Society for Medical Oncology (ESMO) position paper on supportive and palliative care. Ann. Oncol..

[9] Sodergren S.C., Wheelwright S.J., Permyakova N.V., Patel M., Calman L., Smith P.W.F., Din A., Richardson A., Fenlon D., Members of Study Advisory Committee (2019). Supportive care needs of patients following treatment for colorectal cancer: Risk factors for unmet needs and the association between unmet needs and health-related quality of life-results from the ColoREctal Wellbeing (CREW) study. J. Cancer Surviv..

[10] Schouten B., Avau B., E Bekkering G., Vankrunkelsven P., Mebis J., Hellings J., Van Hecke A. (2019). Systematic screening and assessment of psychosocial well-being and care needs of people with cancer. Cochrane Database Syst. Rev..

[11] Tian L., Cao X., Feng X. (2019). Evaluation of psychometric properties of needs assessment tools in cancer patients: A systematic literature review. PLoS ONE.

[12] Boyes A., Girgis A., Lecathelinais C. (2009). Brief assessment of adult cancer patients’ perceived needs: Development and validation of the 34-item Supportive Care Needs Survey (SCNS-SF34). J. Eval. Clin. Pract..

[13] Okuyama T., Akechi T., Yamashita H., Toyama T. (2009). Reliability and validity of the Japanese version of the Short-form Supportive Care Needs Survey questionnaire (SCNS-SF34-J). Psychooncology.

[14] Au A., Lam W.W., Kwong A., Suen D., Tsang J. (2011). Validation of the Chinese version of the short-form Supportive Care Needs Survey Questionnaire (SCNS-SF34-C). Psychooncology.

[15] Schofield P., Gough K., Lotfi-Jam K., Aranda S. (2012). Validation of the Supportive Care Needs Survey-short form 34 with a simplified response format in men with prostate cancer. Psychooncology.

[16] Lehmann C., Koch U., Mehnert A. (2012). Psychometric properties of the German version of the Short-Form Supportive Care Needs Survey Questionnaire (SCNS-SF34-G). Support. Care Cancer.

[17] Brédart A., Kop J.-L., Griesser A.-C., Zaman K., Panes-Ruedin B., Jeanneret W., Delaloye J.-F., Zimmers S., Jacob A., Berthet V. (2012). Validation of the 34-item Supportive Care Needs Survey and 8-item breast module French versions (SCNSSF34-Fr and SCNS-BR8-Fr) in breast cancer patients. Eur. J. Cancer Care.

[18] Li W.W.Y., Lam W.W.T., Shun S.-C., Lai Y.-H., Law W.-L., Poon J., Fielding R. (2013). Psychometric assessment of the Chinese version of the Supportive Care Needs Survey short-form (SCNS-SF34-C) among Hong Kong and Taiwanese Chinese colorectal cancer patients. PLoS ONE.

[19] Jansen F., Witte B.I., van Uden-Kraan C.F., Braspenning A.M., Leemans C.R., Leeuw I.M.V.-D. (2016). The need for supportive care among head and neck cancer patients: Psychometric assessment of the Dutch version of the Supportive Care Needs Survey Short-Form (SCNS-SF34) and the newly developed head and neck cancer module (SCNS-HNC). Support. Care Cancer.

[20] Özbayır T., Geçkil Ö.S., Aslan A. (2017). An Adaptation of the Short-Form Supportive Care Needs Survey Questionnaire (SCNS-SF 34) into Turkish. Eur. J. Breast Health.

[21] Han Y., Zhou Y., Wang J., Zhao Q., Qin H., Fan Y., Song Y., Boyes A., Cui S. (2017). Psychometric testing of the Mandarin version of the 34-item Short-Form Supportive Care Needs Survey in patients with cancer in mainland China. Support. Care Cancer.

[22] Lyu J., Yin L., Cheng P., Li B., Peng S., Yang C., Yang J., Liang H., Jiang Q. (2020). Reliability and validity of the mandarin version of the supportive care needs survey short-form (SCNS-SF34) and the head and neck cancer-specific supportive care needs (SCNS-HNC) module. BMC Health Serv. Res..

[23] Choi E.P.H., Liao Q., Soong I., Chan K.K.L., Lee C.C.Y., Ng A., Sze W.K., Tsang J.W.H., Lee V.H.F., Lam W.W.T. (2020). Measurement invariance across gender and age groups, validity and reliability of the Chinese version of the short-form supportive care needs survey questionnaire (SCNS-SF34). Health Qual. Life Outcomes.

[24] Azman N., Thien L.M., Abdullah M.F.I.L., Mohd Shariff N. (2021). Psychometric Properties of the 34-Item Short-Form Supportive Care Need Survey (SCNS-SF34) Scale in the Malaysian Cancer Healthcare Context. Int. J. Environ. Res. Public Health.

[25] Afework T., Wondimagegnehu A., Alemayehu N., Kantelhardt E.J., Addissie A. (2021). Validity and reliability of the Amharic version of supportive care needs survey—Short form 34 among cancer patients in Ethiopia. BMC Health Serv. Res..

[26] Zeneli A., Fabbri E., Donati E., Tierney G., Pasa S., Berardi M.A., Maltoni M. (2016). Translation of Supportive Care Needs Survey Short Form 34 (SCNS-SF34) into Italian and cultural validation study. Support. Care Cancer.

[27] Moro C., Brunelli C., Miccinesi G., Fallai M., Morino P., Piazza M., Labianca R., Ripamonti C. (2006). Edmonton symptom assessment scale: Italian validation in two palliative care settings. Support. Care Cancer.

[28] McElduff P., Boyes A., Zucca A., Girgis A. The Supportive Care Needs Survey: A Guide to Administration, Scoring and Analysis.

[29] Jackson D.L., Gillapsy J.A., Purc-Stephenson R. (2009). Reporting practices in confirmatory factor analysis: An overview and some recommendations. Psychol. Methods.

[30] Nunnally J.C., Bernstein I.H. (1994). Psychometric Theory.

[31] Hair J.F., Anderson R.E., Tatham R.L., Black W.C. (1998). Multivariate Data Analysis.

[32] Terwee C.B., Bot S.D.M., de Boer M.R., van der Windt D.A.W.M., Knol D.L., Dekker J., Bouter L.M., de Vet H.C.W. (2007). Quality criteria were proposed for measument properties of health status questionnaires. J. Clin. Epidemiol..

[33] Miniotti M., Zeneli A., Bassino S., Pavan S., Ribero S., Leombruni P. (2020). Psychometric assessment of the Italian version of the melanoma module (SCNS-M12-Ita) of the Supportive Care Needs Survey (SCNS-SF34). Tumori.

[34] Doubova S.V., Aguirre-Hernandez R., Gutiérrez-de la Barrera M., Infante-Castañeda C., Pérez-Cuevas R. (2015). Supportive care needs of Mexican adult cancer patients: Validation of the Mexican version of the Short-Form Supportive Care Needs Questionnaire (SCNS-SFM). Support. Care Cancer.

